# Supplementary material for: Outside any therapeutic trial prescription of hydroxychloroquine for hospitalized patients with covid-19 during the first wave of the pandemic: A national inquiry of prescription patterns among French hospitalists
Source: PLoS One. 2022 Jan 21;17(1):e0261843. doi: 10.1371/journal.pone.0261843 (PMC8782345; doi:10.1371/journal.pone.0261843)
Supplement: S1 Fig — (DOCX) [file pone.0261843.s001.docx]

**S1 Fig.** **Correlation between the in-hospital hydroxychloroquine-prescription rate and cumulative in-hospital–mortality rate* according to French region† during the first semester of 2020, ie, the first wave of covid-19. Pearson’s correlation coefficient: 0.049 (95% CI, –0.54 to 0.606).**


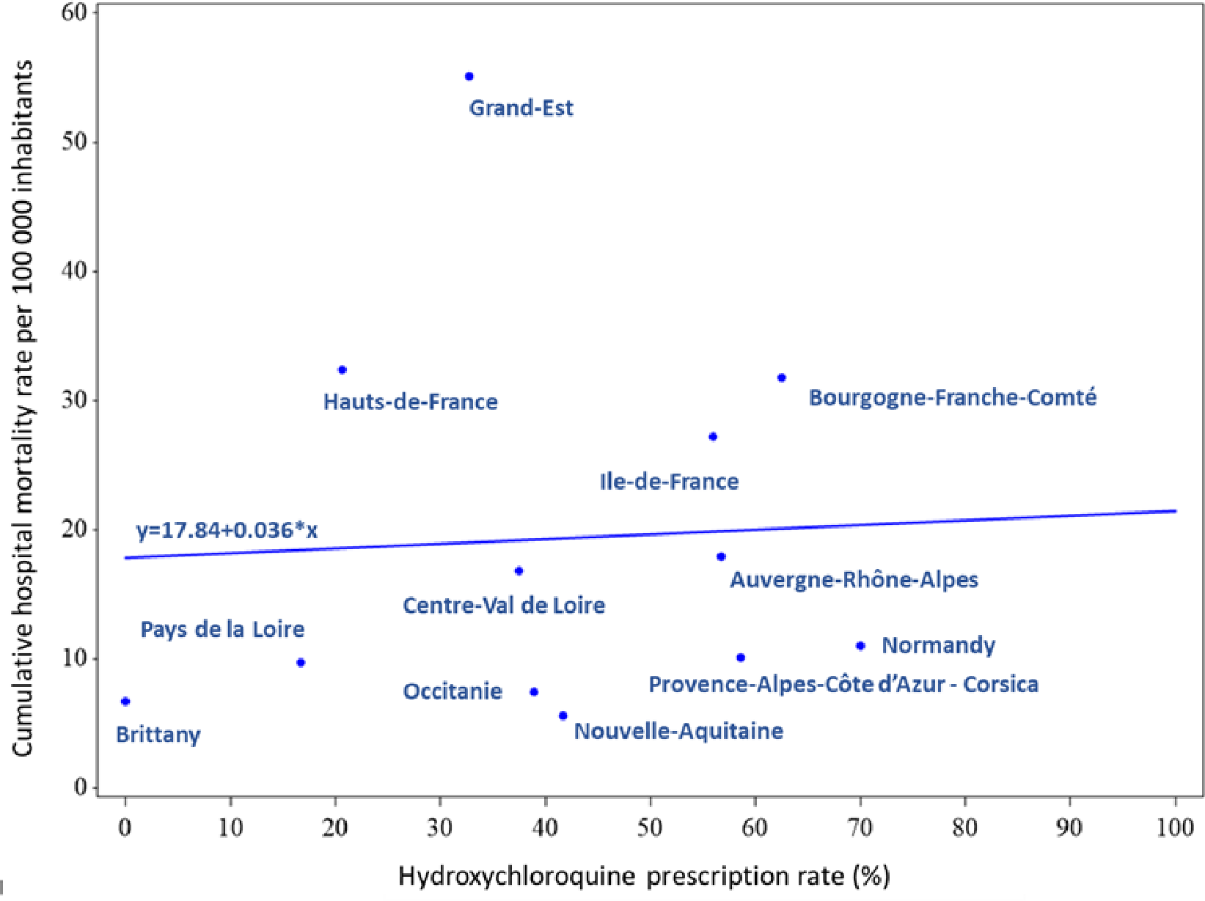


* S4 table.
