# Supplementary material for: Outside any therapeutic trial prescription of hydroxychloroquine for hospitalized patients with covid-19 during the first wave of the pandemic: A national inquiry of prescription patterns among French hospitalists
Source: PLoS One. 2022 Jan 21;17(1):e0261843. doi: 10.1371/journal.pone.0261843 (PMC8782345; doi:10.1371/journal.pone.0261843)
Supplement: S1 Table — (DOCX) [file pone.0261843.s002.docx]

**S1 Table. Observational studies of off-label HCQ prescription for hospitalized covid-19 patients in real-life.**

| **Study (first author)*** | **Country** | **Inclusion period** | **Patients (n)** | **HCQ-treated patients n (%)** |
| --- | --- | --- | --- | --- |
| Alberici F[1] | Italy | 27/02–24/03/2020 | 20 | 20 (100) |
| Alghamdi S[2] | Saudi Arabi | 1/04–31/08/2020 | 775 | 568 (73.3) |
| Allameh SF[3] | Iran | 20/02–17/03/2020 | 905 | 729 (80.6) |
| Arshad S, et al.[4] | USA | 10/03–2/05/2020 | 2541 | 945 (37.2) |
| Awad N, et al.[5] | USA | 20/03–20/04/2020 | 336 | 188 (56) |
| Bartoletti M, et al.[6] | Italy | 22/02–30/06/2020 | 513 | 445 (86.7) |
| Berenguer J, et al.[7] | Spain | Onset†–17/03/2020 | 3995 | 2615 (65.5) |
| Caillard S, et al.[8] | France | 1/03–21/04/2020 | 243 | 60 (24.7) |
| Catteau L, et al.[9] | Belgium | 14/03–24/05/2020 | 8075 | 4542 (56.2) |
| Corist study[10] | Italy | 19/02–23/05/2020 | 3451 | 2633 (76.3) |
| Dubernet A, et al.[11] | France | 11/03–17/04/2020 | 36 | 23 (63.9) |
| Falcone M, et al.[12] | Italy | 4/03–30/04/2020 | 315 | 238 (75.6) |
| Fan X, et al.[13] | USA | 1/04–31/07/2020 | 53,264 | 12,607 (23.7) |
| Fernandez-Ruiz M, et al.[14] | Spain | Onset†–23/03/2020 | 18 | 9 (50) |
| Fried MW, et al.[15] | USA | 15/02–20/04/2020 | 11,721 | 4232 (36.1) |
| Geleris J, et al.[16] | USA | 7/03–8/04/2020 | 1376 | 811 (58.9) |
| Goicoechea M, et al.[17] | Spain | 12/03–10/04/2020 | 36 | 35 (97.2) |
| Gourieux B, et al.[18] | France | 17/03–19/04/2020 | 1193 | 140 (11.7) |
| Guisado-Vasco P, et al.[19] | Spain | 10/03–15/04/2020 | 607 | 558 (91.9) |
| Guner R, et al.[20] | Turkey | 15/03–1/06/2020 | 824 | 604 (73.3) |
| Ip A, et al.[21] | USA | 1/03–22/04/2020 | 2512 | 1914 (76.2) |
| Kim EJ, et al.[22] | USA | 1/03–11/05/2020 | 10,009 | 3270 (32.7) |
| Lammers AJJ, et al.[23] | Netherlands | 28/02–1/04/2020 | 1064 | 566 (53.2) |
| Lauriola M, et al.[24] | Italy | 27/02–20/04/2020 | 317 | 314 (99.1) |
| Lotfy SM.[25] | Egypt | 21/03–8/06/2020 | 202 | 99 (49.0) |
| Loinaz C.[26] | Spain | 15/03–5/05/2020 | 19 | 11 (57.9) |
| Micallef S, et al.[27] | Malta | 1/03–24/04/2020 | 89 | 89 (100) |
| Radovanovic D, et al.[28] | Italy | 7/03–4/04/2020 | 70 | 67 (95.7) |
| Rhodes NJ, et al.[29] | USA | 18/4–8/05/2020 | 352 | 243 (69.0) |
| Rivera DR, et al.[30] | USA | 17/3–26/06/2020 | 2186 | 538 (24.6) |
| Rivera-Azquierdo M, et al.[31] | Spain | 16/3–10/04/2020 | 238 | 215 (90.3) |
| Roig SO, et al.[32] | Spain | 15/3–15/06/2020 | 79 | 67 (84.8) |
| Rosenberg ES, et al.[33] | USA | 15–28/03/2020 | 1438 | 1006 (70) |
| Rosenthal N, et al.[34] | USA | 1/04–31/05/2020 | 35,302 | 18,751 (53.1) |
| Salvador P, et al.[35] | Portugal | 20/3–1/06/2020 | 245 | 121 (49.4) |
| Santos CS, et al.[36] | Spain | 1/03–1/06/2020 | 38 | 31 (81.6) |
| Stefan G, et al.[37] | Romania | 24/03–22/05/2020 | 37 | 22 (59.5) |
| Vahedi E, et al.[38] | Iran | 29/02–23/03/2020 | 60 | 30 (50) |
| Vernaz N, et al.[39] | Switzerland | 26/02–31/05/2020 | 835 | 246 (29.5) |
| **All** |  |  | **145,300** | **59,579 (41.0)** |

*Studies identified after two successive website (<https://pubmed.ncbi.nlm.nih.gov>) searches performed on April 24, 2021 by AB retrieved observational data about HCQ prescription for hospitalized adult covid-19 patients in real life. Search words were: “observational study hydroxychloroquine hospital covid” (search 1), with keywords “covid-19 hospitalization treatment pattern hydroxychloroquine” (search 2). Studies on intensive care unit patients or including the exclusion of certain patients based on received treatments (for example case–control studies) were excluded from the analysis.

†All patients since pandemic onset.

List of references

1. Alberici F, Delbarba E, Manenti C, Econimo L, Valerio F, Pola A, et al. A single center observational study of the clinical characteristics and short-term outcome of 20 kidney transplant patients admitted for SARS-CoV2 pneumonia. Kidney Int. 2020;97: 1083–1088. doi:10.1016/j.kint.2020.04.002

2. Alghamdi S, Barakat B, Berrou I, Alzahrani A, Haseeb A, Hammad MA, et al. Clinical Efficacy of Hydroxychloroquine in Patients with COVID-19: Findings from an Observational Comparative Study in Saudi Arabia. Antibiot Basel Switz. 2021;10. doi:10.3390/antibiotics10040365

3. Allameh SF, Nemati S, Ghalehtaki R, Mohammadnejad E, Aghili SM, Khajavirad N, et al. Clinical Characteristics and Outcomes of 905 COVID-19 Patients Admitted to Imam Khomeini Hospital Complex in the Capital City of Tehran, Iran. Arch Iran Med. 2020;23: 766–775. doi:10.34172/aim.2020.102

4. Arshad S, Kilgore P, Chaudhry ZS, Jacobsen G, Wang DD, Huitsing K, et al. Treatment with hydroxychloroquine, azithromycin, and combination in patients hospitalized with COVID-19. Int J Infect Dis IJID Off Publ Int Soc Infect Dis. 2020;97: 396–403. doi:10.1016/j.ijid.2020.06.099

5. Awad N, Schiller DS, Fulman M, Chak A. Impact of hydroxychloroquine on disease progression and ICU admissions in patients with SARS-CoV-2 infection. Am J Health-Syst Pharm AJHP Off J Am Soc Health-Syst Pharm. 2021;78: 689–696. doi:10.1093/ajhp/zxab056

6. Bartoletti M, Marconi L, Scudeller L, Pancaldi L, Tedeschi S, Giannella M, et al. Efficacy of corticosteroid treatment for hospitalized patients with severe COVID-19: a multicentre study. Clin Microbiol Infect Off Publ Eur Soc Clin Microbiol Infect Dis. 2021;27: 105–111. doi:10.1016/j.cmi.2020.09.014

7. Berenguer J, Ryan P, Rodríguez-Baño J, Jarrín I, Carratalà J, Pachón J, et al. Characteristics and predictors of death among 4035 consecutively hospitalized patients with COVID-19 in Spain. Clin Microbiol Infect. 2020;26: 1525–1536. doi:10.1016/j.cmi.2020.07.024

8. Caillard S, Anglicheau D, Matignon M, Durrbach A, Greze C, Frimat L, et al. An initial report from the French SOT COVID Registry suggests high mortality due to COVID-19 in recipients of kidney transplants. Kidney Int. 2020;98: 1549–1558. doi:10.1016/j.kint.2020.08.005

9. Catteau L, Dauby N, Montourcy M, Bottieau E, Hautekiet J, Goetghebeur E, et al. Low-dose hydroxychloroquine therapy and mortality in hospitalised patients with COVID-19: a nationwide observational study of 8075 participants. Int J Antimicrob Agents. 2020;56: 106144. doi:10.1016/j.ijantimicag.2020.106144

10. COVID-19 RISK and Treatments (CORIST) Collaboration. Use of hydroxychloroquine in hospitalised COVID-19 patients is associated with reduced mortality: Findings from the observational multicentre Italian CORIST study. Eur J Intern Med. 2020;82: 38–47. doi:10.1016/j.ejim.2020.08.019

11. Dubernet A, Larsen K, Masse L, Allyn J, Foch E, Bruneau L, et al. A comprehensive strategy for the early treatment of COVID-19 with azithromycin/hydroxychloroquine and/or corticosteroids: Results of a retrospective observational study in the French overseas department of Réunion Island. J Glob Antimicrob Resist. 2020;23: 1–3. doi:10.1016/j.jgar.2020.08.001

12. Falcone M, Tiseo G, Barbieri G, Galfo V, Russo A, Virdis A, et al. Role of Low-Molecular-Weight Heparin in Hospitalized Patients With Severe Acute Respiratory Syndrome Coronavirus 2 Pneumonia: A Prospective Observational Study. Open Forum Infect Dis. 2020;7: ofaa563. doi:10.1093/ofid/ofaa563

13. Fan X, Johnson BH, Johnston SS, Elangovanraaj N, Coplan P, Khanna R. Evolving Treatment Patterns for Hospitalized COVID-19 Patients in the United States in April 2020-July 2020. Int J Gen Med. 2021;14: 267–271. doi:10.2147/IJGM.S290118

14. Fernández-Ruiz M, Andrés A, Loinaz C, Delgado JF, López-Medrano F, San Juan R, et al. COVID-19 in solid organ transplant recipients: A single-center case series from Spain. Am J Transplant Off J Am Soc Transplant Am Soc Transpl Surg. 2020;20: 1849–1858. doi:10.1111/ajt.15929

15. Fried MW, Crawford JM, Mospan AR, Watkins SE, Munoz Hernandez B, Zink RC, et al. Patient Characteristics and Outcomes of 11,721 Patients with COVID19 Hospitalized Across the United States. Clin Infect Dis Off Publ Infect Dis Soc Am. 2020. doi:10.1093/cid/ciaa1268

16. Geleris J, Sun Y, Platt J, Zucker J, Baldwin M, Hripcsak G, et al. Observational Study of Hydroxychloroquine in Hospitalized Patients with Covid-19. N Engl J Med. 2020;382: 2411–2418. doi:10.1056/NEJMoa2012410

17. Goicoechea M, Sánchez Cámara LA, Macías N, Muñoz de Morales A, Rojas ÁG, Bascuñana A, et al. COVID-19: clinical course and outcomes of 36 hemodialysis patients in Spain. Kidney Int. 2020;98: 27–34. doi:10.1016/j.kint.2020.04.031

18. Gourieux B, Reisz F, Belmas AS, Danion F, Fourtage M, Nai T, et al. Prescribing practices of lopinavir/ritonavir, hydroxychloroquine and azithromycin during the COVID-19 epidemic crisis and pharmaceutical interventions in a French teaching hospital. Eur J Hosp Pharm Sci Pract. 2020. doi:10.1136/ejhpharm-2020-002449

19. Guisado-Vasco P, Valderas-Ortega S, Carralón-González MM, Roda-Santacruz A, González-Cortijo L, Sotres-Fernández G, et al. Clinical characteristics and outcomes among hospitalized adults with severe COVID-19 admitted to a tertiary medical center and receiving antiviral, antimalarials, glucocorticoids, or immunomodulation with tocilizumab or cyclosporine: A retrospective observational study (COQUIMA cohort). EClinicalMedicine. 2020;28: 100591. doi:10.1016/j.eclinm.2020.100591

20. Guner R, Hasanoglu I, Kayaaslan B, Aypak A, Akinci E, Bodur H, et al. Comparing ICU admission rates of mild/moderate COVID-19 patients treated with hydroxychloroquine, favipiravir, and hydroxychloroquine plus favipiravir. J Infect Public Health. 2021;14: 365–370. doi:10.1016/j.jiph.2020.12.017

21. Ip A, Berry DA, Hansen E, Goy AH, Pecora AL, Sinclaire BA, et al. Hydroxychloroquine and tocilizumab therapy in COVID-19 patients-An observational study. PloS One. 2020;15: e0237693. doi:10.1371/journal.pone.0237693

22. Kim EJ, Coppa K, Hirsch JS, Abrahams S, Johnson J, Lesser M, et al. Examination of patient characteristics and hydroxychloroquine use based on the US Food and Drug Administration’s recommendation: a cross-sectional analysis in New York. BMJ Open. 2021;11: e042965. doi:10.1136/bmjopen-2020-042965

23. Lammers AJJ, Brohet RM, Theunissen REP, Koster C, Rood R, Verhagen DWM, et al. Early hydroxychloroquine but not chloroquine use reduces ICU admission in COVID-19 patients. Int J Infect Dis IJID Off Publ Int Soc Infect Dis. 2020;101: 283–289. doi:10.1016/j.ijid.2020.09.1460

24. Lauriola M, Pani A, Ippoliti G, Mortara A, Milighetti S, Mazen M, et al. Effect of Combination Therapy of Hydroxychloroquine and Azithromycin on Mortality in Patients With COVID-19. Clin Transl Sci. 2020;13: 1071–1076. doi:10.1111/cts.12860

25. Lotfy SM, Abbas A, Shouman W. Use of Hydroxychloroquine in Patients with COVID-19: A Retrospective Observational Study. Turk Thorac J. 2021;22: 62–66. doi:10.5152/TurkThoracJ.2021.20180

26. Loinaz C, Marcacuzco A, Fernández-Ruiz M, Caso O, Cambra F, San Juan R, et al. Varied clinical presentation and outcome of SARS-CoV-2 infection in liver transplant recipients: Initial experience at a single center in Madrid, Spain. Transpl Infect Dis Off J Transplant Soc. 2020;22: e13372. doi:10.1111/tid.13372

27. Micallef S, Piscopo TV, Casha R, Borg D, Vella C, Zammit M-A, et al. The first wave of COVID-19 in Malta; a national cross-sectional study. PloS One. 2020;15: e0239389. doi:10.1371/journal.pone.0239389

28. Radovanovic D, Pini S, Franceschi E, Pecis M, Airoldi A, Rizzi M, et al. Characteristics and outcomes in hospitalized COVID-19 patients during the first 28 days of the spring and autumn pandemic waves in Milan: An observational prospective study. Respir Med. 2021;178: 106323. doi:10.1016/j.rmed.2021.106323

29. Rhodes NJ, Dairem A, Moore WJ, Shah A, Postelnick MJ, Badowski ME, et al. Multicenter point prevalence evaluation of the utilization and safety of drug therapies for COVID-19 at the onset of the pandemic timeline in the United States. Am J Health-Syst Pharm AJHP Off J Am Soc Health-Syst Pharm. 2021;78: 568–577. doi:10.1093/ajhp/zxaa426

30. Rivera DR, Peters S, Panagiotou OA, Shah DP, Kuderer NM, Hsu C-Y, et al. Utilization of COVID-19 Treatments and Clinical Outcomes among Patients with Cancer: A COVID-19 and Cancer Consortium (CCC19) Cohort Study. Cancer Discov. 2020;10: 1514–1527. doi:10.1158/2159-8290.CD-20-0941

31. Rivera-Izquierdo M, Valero-Ubierna MDC, R-delAmo JL, Fernández-García MÁ, Martínez-Diz S, Tahery-Mahmoud A, et al. [Therapeutic agents tested in 238 COVID-19 hospitalized patients and their relationship with mortality]. Med Clin (Barc). 2020;155: 375–381. doi:10.1016/j.medcli.2020.06.025

32. Ortonobes Roig S, Soler-Blanco N, Torrente Jiménez I, Van den Eynde Otero E, Moreno-Ariño M, Gómez-Valent M. [Clinical and pharmacological data in COVID-19 hospitalized nonagenarian patients]. Rev Espanola Quimioter Publicacion Of Soc Espanola Quimioter. 2021;34: 145–150. doi:10.37201/req/130.2020

33. Rosenberg ES, Dufort EM, Udo T, Wilberschied LA, Kumar J, Tesoriero J, et al. Association of Treatment With Hydroxychloroquine or Azithromycin With In-Hospital Mortality in Patients With COVID-19 in New York State. JAMA. 2020;323: 2493–2502. doi:10.1001/jama.2020.8630

34. Rosenthal N, Cao Z, Gundrum J, Sianis J, Safo S. Risk Factors Associated With In-Hospital Mortality in a US National Sample of Patients With COVID-19. JAMA Netw Open. 2020;3: e2029058. doi:10.1001/jamanetworkopen.2020.29058

35. Salvador P, Oliveira P, Costa T, Fidalgo M, Neto R, Silva ML, et al. Clinical Features and Prognostic Factors of 245 Portuguese Patients Hospitalized With COVID-19. Cureus. 2021;13: e13687. doi:10.7759/cureus.13687

36. Santos CS, Morales CM, Álvarez ED, Castro CÁ, Robles AL, Sandoval TP. Determinants of COVID-19 disease severity in patients with underlying rheumatic disease. Clin Rheumatol. 2020;39: 2789–2796. doi:10.1007/s10067-020-05301-2

37. Stefan G, Mehedinti AM, Andreiana I, Zugravu AD, Cinca S, Busuioc R, et al. Clinical features and outcome of maintenance hemodialysis patients with COVID-19 from a tertiary nephrology care center in Romania. Ren Fail. 2021;43: 49–57. doi:10.1080/0886022X.2020.1853571

38. Vahedi E, Ghanei M, Ghazvini A, Azadi H, Izadi M, Panahi Y, et al. The clinical value of two combination regimens in the Management of Patients Suffering from Covid-19 pneumonia: a single centered, retrospective, observational study. Daru J Fac Pharm Tehran Univ Med Sci. 2020;28: 507–516. doi:10.1007/s40199-020-00353-w

39. Vernaz N, Agoritsas T, Calmy A, Gayet-Ageron A, Gold G, Perrier A, et al. Early experimental COVID-19 therapies: associations with length of hospital stay, mortality and related costs. Swiss Med Wkly. 2020;150: w20446. doi:10.4414/smw.2020.20446
