# Supplementary material for: Outside any therapeutic trial prescription of hydroxychloroquine for hospitalized patients with covid-19 during the first wave of the pandemic: A national inquiry of prescription patterns among French hospitalists
Source: PLoS One. 2022 Jan 21;17(1):e0261843. doi: 10.1371/journal.pone.0261843 (PMC8782345; doi:10.1371/journal.pone.0261843)
Supplement: S2 Table — (DOCX) [file pone.0261843.s003.docx]

**S2 Table. Comparison of participants’ specialties.**

|  | **Prescription of HCQ for Covid-19 patients** | | | |
| --- | --- | --- | --- | --- |
| **Specialty*** | **Total, n (%)** | **Yes, n (%)** | **No, n (%)** | **P value†** |
| Cardiology |  |  |  | 0.0918 (F) |
| n | 400 | 181 | 219 |  |
| Yes | 3 | 3 (1.7) | 0 (0) |  |
| No | 397 | 178 (98.3) | 219 (100.0) |  |
| Geriatrics |  |  |  | 0.1405 (C) |
| n | 400 | 181 | 219 |  |
| Yes | 15 | 4 (2.2) | 11 (5.0) |  |
| No | 385 | 177 (97.8) | 208 (95.0) |  |
| Hematology–Oncology |  |  |  | 0.1336 (F) |
| n | 400 | 181 | 219 |  |
| Yes | 7 | 1 (0.6) | 6 (2.7) |  |
| No | 393 | 180 (99.4) | 213 (97.3) |  |
| Infectious Diseases |  |  |  | 0.566 (C) |
| n | 400 | 181 | 219 |  |
| Yes | 172 | 75 (41.4) | 97 (44.3) |  |
| No | 128 | 106 (58.6) | 122 (55.7) |  |
| Internal medicine |  |  |  | 0.002 (C) |
| n | 400 | 181 | 219 |  |
| Yes | 165 | 90 (49.7) | 75 (34.2) |  |
| No | 135 | 91 (50.3) | 144 (65.8) |  |
| Pneumology |  |  |  | 0.1960 (F) |
| n | 400 | 181 | 219 |  |
| Yes | 10 | 7 (3.9) | 3 (1.4) |  |
| No | 390 | 174 (96.1) | 216 (98.6) |  |
| General Medicine |  |  |  | 0.019 (C) |
| n | 400 | 181 | 219 |  |
| Yes | 37 | 10 (5.5) | 27 (12.3) |  |
| No | 363 | 171 (94.5) | 192 (87.7) |  |
| Rheumatology |  |  |  | 1.000 (F) |
| n | 400 | 181 | 219 |  |
| Yes | 4 | 2 (1.1) | 2 (0.9) |  |
| No | 396 | 179 (98.9) | 217 (99.1) |  |
| Vascular Medicine |  |  |  | 0.6620 (F) |
| n | 400 | 181 | 219 |  |
| Yes | 5 | 3 (1.7) | 2 (1.4) |  |
| No | 395 | 178 (98.3) | 217 (98.6) |  |
| Other specialties |  |  |  | 0.2264 (C) |
| n | 400 | 181 | 219 |  |
| Yes | 39 | 10 (5.5) | 19 (8.7) |  |
| No | 371 | 171 (94.5) | 200 (91.3) |  |

†(C) Chi-2 test; (F) Fisher’s exact test.

* Some physicians declared multiple specialties.
