# Supplementary material for: Outside any therapeutic trial prescription of hydroxychloroquine for hospitalized patients with covid-19 during the first wave of the pandemic: A national inquiry of prescription patterns among French hospitalists
Source: PLoS One. 2022 Jan 21;17(1):e0261843. doi: 10.1371/journal.pone.0261843 (PMC8782345; doi:10.1371/journal.pone.0261843)
Supplement: S3 Table — (DOCX) [file pone.0261843.s004.docx]

**S3 Table. HCQ-prescription determinants: sources of information on HCQ to treat covid-19**

|  | | **Univariate analysis*** | |
| --- | --- | --- | --- |
| **Sources of information on HCQ** | **aOR [95% CI]** | | **P value** |
| Learned societies |  | |  |
| No | 1 | |  |
| Yes | 1.01 [0.67 to 1.54] | | 0.9526 |
| Medical journals (peer reviewed) |  | |  |
| No | 1 | |  |
| Yes | 1.21 [0.72 to 2.04] | | 0.4798 |
| Medical journals (no peer review) |  | |  |
| No | 1 | |  |
| Yes | 1.18 [0.74 to 1.88] | | 0.4990 |
| Hospital colleagues |  | |  |
| No | 1 | |  |
| Yes | 1.6 [1.08 to 2.38] | | 0.0201 |
| Hospital information sheet |  | |  |
| No | 1 | |  |
| Yes | 1.77 [1.13 to 2.78] | | **0.00127** |
| Web |  | |  |
| No | 1 | |  |
| Yes | 0.76 [0.49 to 1.17] | | 0.2089 |
| Social media |  | |  |
| No | 1 | |  |
| Yes | 1.15 [0.56 to 2.35] | | 0.6969 |

*****First, univariate analyses (p<0.2) selected potential explanatory variables that were then entered into the multivariate model (stepwise method with entry/stay significance levels of 0.2/0.05). Results are expressed as adjusted odd ratios (aOR) [ 95% confidence interval (CI)]. Multivariate analysis did not retain any source of information on HCQ as a factor independently associated with HCQ prescription.
