# Supplementary material for: Outside any therapeutic trial prescription of hydroxychloroquine for hospitalized patients with covid-19 during the first wave of the pandemic: A national inquiry of prescription patterns among French hospitalists
Source: PLoS One. 2022 Jan 21;17(1):e0261843. doi: 10.1371/journal.pone.0261843 (PMC8782345; doi:10.1371/journal.pone.0261843)
Supplement: S4 Table — (DOCX) [file pone.0261843.s005.docx]

**S4 table. Cumulative in-hospital–mortality rate per 100,000 inhabitants linked to covid-19 on May 7, 2020 in France.**

| **French regions†** | **Cumulative hospital mortality linked to covid-19‡** | **Population (INSEE 2020)** | **Peak mortality rate per 100,000 inhabitants** |
| --- | --- | --- | --- |
| Auvergne-Rhône-Alpes | 1437 | 8 032 377 | **17.9** |
| Bourgogne-Franche-Comté | 884 | 2 783 039 | **31.8** |
| Brittany | 223 | 3 340 379 | **6.7** |
| Centre-Val de Loire | 429 | 2 559 073 | **16.8** |
| Grand Est | 3037 | 5 511 747 | **55.1** |
| Hauts-de-France | 1930 | 5 962 662 | **32.4** |
| Ile-de-France | 3338 | 12 278 210 | **27.2** |
| Normandy | 365 | 3 303 500 | **11.0** |
| Nouvelle Aquitaine | 336 | 5 999 982 | **5.6** |
| Occitanie | 436 | 5 924 858 | **7.4** |
| Pays de Loire | 368 | 3 801 797 | **9.7** |
| Provence-Alpes-Côte d’Azur and Corsica | 545 | 5 400 330 | **10.1** |
| Metropolitan France | 16060 | 64 897 954 | **20.5** |

†Northwest: Normandy, Brittany, Centre-Val de Loire and Pays de la Loire; Northeast: Bourgogne-Franche-Comté, Grand Est and Hauts-de-France, Southwest: Nouvelle-Aquitaine and Occitanie, Southeast: Auvergne-Rhône-Alpes, Provence-Alpes-Côte d’Azur and Corsica.

‡Cumulative in-hospital mortality linked to covid-19 on May 7, 2020

Auvergne: <https://www.santepubliquefrance.fr/regions/auvergne-rhone-alpes/documents/bulletin-regional/2020/covid-19-point-epidemiologique-en-auvergne-rhone-alpes-du-7-mai-2020>

Bourgogne: https://www.santepubliquefrance.fr/regions/bourgogne-franche-comte/documents/bulletin-regional/2020/covid-19-point-epidemiologique-en-bourgogne-et-franche-comte-du-7-mai-2020

Brittany: <https://www.santepubliquefrance.fr/regions/bretagne/documents/bulletin-regional/2020/covid-19-point-epidemiologique-en-bretagne-du-7-mai-2020>

Centre-Val de Loir : <https://www.santepubliquefrance.fr/regions/centre-val-de-loire/documents/bulletin-regional/2020/covid-19-point-epidemiologique-en-centre-val-de-loire-du-7-mai-2020>

Grand Est: https://www.santepubliquefrance.fr/regions/grand-est/documents/bulletin-regional/2020/covid-19-point-epidemiologique-en-grand-est-du-6-mai-2020

Hauts-de-France: https://www.santepubliquefrance.fr/regions/hauts-de-france/documents/bulletin-regional/2020/covid-19-point-epidemiologique-en-hauts-de-france-du-7-mai-2020

Ile-de-France: https://www.santepubliquefrance.fr/regions/ile-de-france/documents/bulletin-regional/2020/covid-19-point-epidemiologique-en-ile-de-france-du-7-mai-2020

Normandy : <https://www.santepubliquefrance.fr/regions/normandie/documents/bulletin-regional/2020/covid-19-point-epidemiologique-en-normandie-du-7-mai-2020>

Nouvelle Aquitain : <https://www.santepubliquefrance.fr/regions/nouvelle-aquitaine/documents/bulletin-regional/2020/covid-19-point-epidemiologique-en-nouvelle-aquitaine-du-7-mai-2020>

Occitanie: https://www.santepubliquefrance.fr/regions/nouvelle-aquitaine/documents/bulletin-regional/2020/covid-19-point-epidemiologique-en-nouvelle-aquitaine-du-7-mai-2020

Pays de Loire: https://www.santepubliquefrance.fr/regions/pays-de-la-loire/documents/bulletin-regional/2020/covid-19-point-epidemiologique-en-pays-de-la-loire-du-7-mai-2020

Provence-Alpes-Côte d’Azur-Corsica: https://www.santepubliquefrance.fr/regions/provence-alpes-cote-d-azur-et-corse/documents/bulletin-regional/2020/covid-19-point-epidemiologique-en-paca-du-6-mai-2020

Institut National de la Statistique et des Etudes Economiques (INSEE): https://www.insee.fr/fr/statistiques/1893198
