# Supplementary material for: Outside any therapeutic trial prescription of hydroxychloroquine for hospitalized patients with covid-19 during the first wave of the pandemic: A national inquiry of prescription patterns among French hospitalists
Source: PLoS One. 2022 Jan 21;17(1):e0261843. doi: 10.1371/journal.pone.0261843 (PMC8782345; doi:10.1371/journal.pone.0261843)
Supplement: S1 Appendix — (DOCX) [file pone.0261843.s006.docx]

**S1 Appendix. Inquiry questionnaire (French)**

**Etude Cov-Hyd (Hyd**roxychloroquine au cours du **Cov**id-19**)**

**Section 1**

Vous êtes médecin sénior et avez pris en charge un ou des patients atteints du virus SARS-CoV2 responsable de la maladie "Covid-19" en hospitalisation. Nous vous proposons de répondre à cette enquête sur la question de la prescription d'hydroxychloroquine (HCQ) chez les patients atteints de Covid-19. Les réponses sont traitées de façon anonyme. Sauf mention contraire, les questions portent sur la prescription d'HCQ chez les patients hospitalisés hors essai thérapeutique prospectif randomisé. Cette enquête s’adresse autant aux médecins ayant prescrit de l’HCQ qu’à ceux qui n’en n’ont pas prescrit.

Acceptez-vous de répondre à cette enquête (temps estimé de 5 environ) ? *

Oui

Non.

**Section 2**

Caractéristiques des participants

Nous vous remercions pour votre participation.

Nous sommes aujourd'hui le *

Vous êtes ? *

Une femme, Un homme, Je ne souhaite pas le préciser

Vous exercez depuis ? *

0-4 ans, 5-9 ans, 10-19 ans, 20-29 ans, 30 ans ou plus

Votre spécialité est la : *

Médecine interne, Cardiologie, Gériatrie, Hématologie – Oncologie, Infectiologie, Médecine Vasculaire, Pneumologie, Rhumatologie, Autres

Vous avez pris en charge des patients Covid-19 dans un service appartenant à *

un hôpital public universitaire (CHU)

un hôpital public non universitaire

un hôpital privé (ou clinique privée) à but non lucratif

un hôpital privé (ou clinique privée) à but lucratif

Vous travaillez dans la région suivante *

Avez-vous déjà prescrit de l'hydroxychloroquine avant l'épidémie de Covid-19 dans une autre indication ? *

Oui, Non, Je ne sais pas

__________________________________________________________________________________________

**Section 3**

Procédures de prescription de l'HCQ dans le service hors essai thérapeutique prospectif randomisé

Existe-t-il une procédure commune de prescription de l'HCQ dans votre service ? *

Oui *(passer à la section suivante)*

Non *(passer à la section 7)*

Je ne sais pas *(passer à la section 7)*

**Section 4**

Procédures de prescription de l'HCQ

Dans ce cas, la procédure est *

de ne jamais prescrire d'HCQ dans le service ? *(passer à la section 7)*

de proposer à tous les malades hospitalisés de recevoir de l'HCQ *(passer à la section 6)*

de proposer à certains malades hospitalisés de recevoir de l'HCQ selon des critères prédéfinis (*passer à la section 6)*

de ne prescrire de l'HCQ qu'aux patients qui en font la demande *(passer à la section 6)*

de laisser l'initiative de prescrire de l'HCQ à l'appréciation de chaque praticien ? *(passer à la section 7)*

de discuter de façon collégiale au cas par cas des patients à qui de l'HCQ va être proposée *(passer à la section suivante)*

je ne sais pas (passer à la section 7)

**Section 5**

Procédures de prescription de l'HCQ

La discussion collégiale a lieu *

lors d'un staff, lors des visites "au lit du malade", par téléphone, autres (*passer la section suivante)*

**Section 6**

Application de la décision collégiale de prescription d'HCQ

En cas de décision collégiale de prescrire de l'HCQ à l'un de vos patients, proposez-vous effectivement de l'HCQ à votre patient ?

Toujours, Souvent, Parfois, Rarement, Jamais

*Passer à la section suivante*

**Section 7**

Procédure d'information du patient

Existe-t-il une procédure d'information des patients Covid-19 sur la prescription d'HCQ dans cadre de leur infection ? *

Non *(passer à la section 9)*

Oui et destinée uniquement aux patients à qui l'HCQ est proposée *(passer à la section suivante)*

Oui et destinée à tous les patients que l'indication de l'HCQ soit retenue ou pas *(passer à la section suivante)*

Je ne sais pas *(passer à la section 9)*

**Section 8**

Procédure d'information du patient (question complémentaire en cas de procédure)

Est-ce une information formalisée sous forme ? *

écrite

orale

*Passer à la section suivante*

*__________________________________________________________________________________________*

**Section 9**

Prescription d'HCQ pour les patients hospitalisés pour Covid-19

Avez-vous déjà prescrit un traitement par HCQ chez un ou plusieurs patient(s) Covid-19 lorsque vous en aviez la charge (hors essai prospectif randomisé) ? *

Oui *(passer à la section suivante)*

Non *(passer à la section 26)*

Je ne sais pas *(passer à la section 26)*

__________________________________________________________________________________________

**Section 10 (HYDROXYCHLOROQUINE +)**

Prescription d'HCQ pour les patients hospitalisés pour Covid-19

S'agit-il d'un traitement par HCQ initié chez un (ou plusieurs) patient(s) qui étai(en)t sous votre responsabilité lors de son instauration ? *

Oui *(passer à la section suivante)*

Non (*passer à la section 17)*

**Section 11**

Prescription d'HCQ pour les patients hospitalisés pour Covid-19

Dans ce cas, qui a été à l'initiative de la discussion d'éventuellement prescrire de l'HCQ ? *

Vous, le patient, l'entourage du patient, un collègue, la procédure de service en vigueur, un avis collégial

Autre :

*Passer à la section suivante*

**Section 12**

Prescription d'HCQ pour les patients hospitalisés pour Covid-19

Le mode final de décision de prescrire ou de ne pas prescrire de l'HCQ est-il ? *

Collégial, Individuel

*Passer à la section suivante*

**Section 13**

Prescription d'HCQ pour les patients hospitalisés pour Covid-19

Avez-vous proposé de recevoir de l'HCQ (hors contre-indication) ? *

A tous vos patients *(passer à la section 16)*

Uniquement à certains d'entre eux *(passer à la section suivante)*

**Section 14**

Prescription d'HCQ pour les patients hospitalisés pour Covid-19

Dans ce cas, avez-vous prescrit l'HCQ selon l'autonomie du patient ? *

Oui plutôt chez les patients autonomes

Oui plutôt chez les patients non autonomes

Non, pas en fonction de leur autonomie

Je ne sais pas

*Passer à la section suivante*

Avez-vous prescrit l'HCQ selon l'existence de comorbidités ? (diabète, terrain vasculaire, surpoids/obésité, BPCO ....) *

Oui plutôt chez les patients avec comorbidités

Oui plutôt chez les patients sans comorbidités

Non, pas en fonction de leur comorbidités

Je ne sais pas

Avez-vous prescrit l'HCQ selon leur statut réanimatoire ou non réanimatoire ? *

Oui plutôt chez les patients "réanimatoires"

Oui plutôt chez les patients "non réanimatoires"

Non, pas en fonction de leur statut "réanimatoire"

Je ne sais pas

Avez-vous prescrit l'HCQ selon l'âge des patients ? *

Oui, Non, Je ne sais pas

Passer à la section suivante

Avez-vous prescrit l'HCQ selon les caractéristiques de la maladie Covid-19 (sévérité, moment par rapport au début des symptômes, tendance évolutive) ? *

Oui *(passer à la section suivante)*

Non *(passer à la section 16)*

Je ne sais pas *(passer à la section 16)*

**Section 15**

Prescription d'HCQ pour les patients hospitalisés pour Covid-19

Dans ce cas, est-ce plutôt ? *

à un stade précoce (par exemple avant 7 jours du début des symptômes)

à un stade intermédiaire (par exemple entre le 7è et le 10è jour à partir du début des symptômes)

à un stade plus tardif (par exemple après le 10è jour du début des symptômes)

en cas de forme bénigne

en cas de forme de gravité intermédiaire

en cas de forme grave

en cas de tendance à l'aggravation

en cas de tendance à la stabilité

en cas de tendance à l'amélioration

je ne sais pas

*Passer à la section suivante*

**Section 16**

Critères de prescription d'HCQ au cours du Covid-19

Pour quels motifs avez-vous prescrit de l'HCQ à vos patients Covid-19 ? (plusieurs réponses possibles) *

C'est un médicament ancien avec un profil de tolérance connu et favorable

Sa prescription est possible grâce un décret ministériel

J'applique l'avis de la décision collégiale

Il s'agit d'une demande du patient ou de son entourage

La maladie Covid-19 est (potentiellement) très grave

L'HCQ semble efficace au cours du Covid-19

L'HCQ est un médicament peu cher et disponible

C'était la seule option thérapeutique à ma disposition (absence d'alternative)

Par crainte des conséquences médico-légales

Par crainte du regard de mes confrères de mon service ou de mon hôpital

L'efficacité de l'HCQ au cours du Covid-19 n'est pas certaine mais les règles habituelles d'évaluation des médicaments ne s'appliquent pas en situation d'urgence sanitaire

C'est un traitement recommandé par des confrères avec qui j'en ai discuté

C'est un traitement recommandé par des "autorités médicales"

Il me parait difficile de résister à la pression médiatique et/ou sociétale

Le patient a déjà reçu de l'HCQ avec une bonne tolérance pour une autre indication

Voyez-vous une autre raison ? si oui, merci de préciser ci-dessous

*Passer à la section 18*

__________________________________________________________________________________________

**Section 17 (hydroxychloroquine +/-)**

Motifs de l'abstention d'HCQ au cours du Covid-19

Pour quelle(s) raison(s) n'avez-vous pas initié vous-même un traitement par HCQ à l'un de vos patients lors de l'épidémie de Covid-19 (hors essai thérapeutique) ? *

Absence d'indication selon les données actuelles disponibles de la science médicale

Vous ne vous êtes pas posé la question d'en prescrire

Une discussion collégiale n'était pas organisée dans votre service

Vous êtes opposé à une prescription hors AMM

Vous jugez non éthique de prescrire un traitement non validé en dehors d'essais thérapeutiques

Absence de recommandations officielles en faveur de la prescription d'HCQ (Sociétés Savantes, Académie de Médecine, Ordre)

Crainte des conséquences médico-légales

Crainte que l'HCQ puisse aggraver la maladie Covid-19

Crainte des effets secondaires potentiels

Refus du patient ou de son entourage

Crainte des réactions ou des avis de mes confrères

Vous n'interveniez pas dans la prise en charge "spécifique Covid-19" de vos malades

Aucun de vos patients ne remplissait les critères de prescription prévus dans votre service

Autre

*Passer à la section suivante*

__________________________________________________________________________________________

**Section 18**

Transfert d'un patient sous HCQ dans votre unité

Dans le cas d'un patient transféré sous HCQ dans votre secteur, que faites-vous concernant la prescription d'HCQ (en l'absence de contre-indication) ? *

Poursuite de l'HCQ selon les modalités des confrères l'ayant initié

Poursuite de l'HCQ en s'assurant de l'accord du patient et/ou de son entourage (personne de confiance/tuteur)

Rediscussion de l'indication de l'HCQ

Arrêt de l'HCQ en s'assurant de l'accord du patient et/ou de son entourage (personne de confiance/tuteur)

Arrêt de l'HCQ étant en désaccord avec son utilisation au cours du Covid-19

*Passer à la section suivante*

**Section 19**

Questions complémentaires en cas de prescription d'HCQ au cours du Covid-19

Quel était le protocole de prescription de l'HCQ (hors essai prospectif randomisé) ? *

Utilisation d'une dose de charge

Prescription en monothérapie

En association à l'azithromycine

Je ne sais pas

Aucune de ces réponses

Avez-vous constaté des effets indésirables liés à l'HCQ chez vos patients ? *

Oui *(passer à la section suivante)*

Non *(passer à la section 21)*

Je ne sais pas *(passer à la section 21)*

**Section 20**

Effets indésirables de l'HCQ

Pour quelle proportion de vos patients ? * (menu déroulant)

Quels étaient les effets indésirables que vous avez observés ? *

Allongement du QT corrigé

Troubles du rythmes cardiaques (torsades de pointes, tachycardie ventriculaires)

Troubles digestifs (douleur abdominale, nausées, diarrhées, vomissements)

Lésions dermatologiques bénignes

Céphalées

Manifestations allergiques graves (Stevens-Johnson, Lyell ...)

Autres

Je ne sais pas

Cela a-t-il conduit parfois à interrompre le traitement par HCQ ? *

Oui, Non, Je ne sais pas

Cela a-t-il eu les conséquences suivantes pour le patient ? *

Prolongation d'hospitalisation

Transfert dans un autre service

Séquelles

Décès

Je ne sais pas

*Passer à la section suivante*

**Section 21**

Prescription d'HCQ vous paraissant non justifiée

Vous est-il arrivé de prescrire de l'HCQ à l'un de vos patients alors que cette prescription ne vous paraissait pas justifiée ? *

Oui (passer à la section suivante)

Non (passer à la section 23)

Je ne sais pas (passer à la section 23)

*Passer à la section suivante*

**Section 22**

Prescription d'HCQ vous paraissant non justifiée

Dans ce cas, était-ce ? *

Une prescription initiée dans un autre service et que vous avez poursuivie

Une prescription décidée dans votre service lors d'une discussion collégiale

Une prescription décidée dans votre service par un collègue

Je ne sais pas

*Passer à la section suivante*

**Section 23**

Questions complémentaires pour les prescripteurs d'HCQ (hors essai prospectif randomisé)

Comment estimez-vous le stress engendré par le fait de prescrire de l'HCQ (au cours du Covid-19) qui est un traitement dont le rapport bénéfice/risque n'est pas établi au regard des critères habituels de la science médicale ? *

Echelle de 0 à 10 (aucun stress ------stress maximal imaginable)

A propos de vos prescriptions d'HCQ (hors essai prospectif randomisé), avez-vous prévu de communiquer votre expérience ? (article, congrès) *

Non *(passer à la section 25)*

Oui *(passer à la section suivante)*

Je ne sais pas *(passer à la section 25)*

**Section 24**

Questions complémentaires pour les prescripteurs d'HCQ

Etait-ce prévu à priori avant de débuter vos prescriptions ? *

Oui

Non

Je ne sais pas

Dans ce cas, est-ce sous forme de ? *

Cas clinique

Etude rétrospective de cohorte

Etude d'évaluation des pratiques professionnelles (EPP)

Si vous prévoyez une étude rétrospective de cohorte, comporte-t-elle un groupe contrôle ? *

Je ne prévois pas d'étude de cohorte

Oui

Non

*Passer à la section suivante*

**Section 25**

Modification des attitudes de prescription de l'HCQ

Vous avez prescrit de l'HCQ au cours du Covid-19. Votre attitude de prescription a-t-elle changé au cours de l'épidémie ? *

Non

Oui dans le sens d'un arrêt des prescriptions

Oui dans le sens d'une diminution de prescription

Oui dans le sens d'une augmentation de prescription

Oui vous avez finalement commencé à en prescrire

Je ne sais pas

*Passer à la section 34*

__________________________________________________________________________________________

**Section 26 (HYDROXYCHLOROQUINE -)**

Absence de prescription d'HCQ au cours du Covid-19

Pour quelle(s) raison(s) n'avez-vous pas prescrit d'HCQ au cours de l'épidémie Covid-19 (hors essai prospectif randomisé) ? *

Absence d'indication selon les données actuelles disponibles de la science médicale

Vous ne vous êtes pas posé la question d'en prescrire

Une discussion collégiale n'était pas organisée dans votre service

Vous êtes opposé à une prescription hors AMM

Vous jugez non éthique de prescrire un traitement non validé en dehors d'essais thérapeutiques

Absence de recommandations officielles en faveur de la prescription d'HCQ (Sociétés Savantes, Académie de Médecine, Ordre)

Crainte des conséquences médico-légales

Refus du patient ou de son entourage

Crainte des réactions ou des avis de mes confrères

Crainte que l'HCQ puisse aggraver la maladie Covid-19

Crainte des effets secondaires potentiels

Vous n'interveniez pas dans la prise en charge "spécifique Covid-19" de vos malades

Aucun de vos patients ne remplissait les critères prévus dans votre service par vous ou collégialement

Autre

*Passer à la section suivante*

**Section 27**

Absence de prescription d'HCQ au cours du Covid-19

Etes-vous dans la situation où tous vos patients étaient inclus dans des essais prospectifs randomisés ? *

Oui *(passer à la section suivante)*

Non *(passer à la section 31)*

**Section 28**

Inclusion dans des essais thérapeutiques

Si tous vos patients n'avaient pas été inclus dans des essais prospectifs randomisés, auriez-vous prescrit potentiellement de l'HCQ ? *

Oui *(passer à la section 30)*

Non *(passer à la section suivante)*

Je ne sais pas *(passer à la section 34)*

**Section 29**

Je n'aurais pas prescrit de l'HCQ

Pourquoi ? *

Absence d'indication selon les données actuelles disponibles de la science médicale

Une discussion collégiale n'était pas organisée dans votre service

Vous êtes opposé à une prescription hors AMM

Vous jugez non éthique de prescrire un traitement non validé en dehors d'un essai thérapeutique

Absence de recommandations officielles en faveur de la prescription d'HCQ (Sociétés Savantes, Académie de Médecine, Ordre)

Crainte des conséquences médico-légales

Refus du patient ou de son entourage

Crainte que l'HCQ puisse aggraver la maladie Covid-19

Crainte des effets secondaires potentiels de l’HCQ

Crainte des réactions ou des avis de mes confrères

Vous l'interveniez pas dans la prise en charge "spécifique Covid-19" de vos malades

Autre

*Passer à la section 31*

**Section 30**

J'aurais prescrit de l'HCQ

Vous en auriez prescrit de l'HCQ chez vos patients Covid. Pour quelle(s) raison(s) ? (plusieurs réponses possibles) *

C'est un médicament ancien avec un profil de tolérance connu et favorable

Sa prescription est possible grâce un décret ministériel

Je n'aurais fait qu'appliquer l'avis de la décision collégiale

En cas de demande du patient ou de son entourage

La maladie Covid-19 est (potentiellement) très grave

L'HCQ semble efficace au cours du Covid-19

L'HCQ est un médicament peu cher et disponible

C'était la seule option thérapeutique à ma disposition (absence d'alternative)

Crainte des conséquences médico-légales

Crainte du regard de mes confrères de mon service ou de mon hôpital

L'efficacité de l'HCQ au cours du Covid-19 n'est pas prouvée mais les règles habituelles d'évaluation des médicaments ne s'appliquent pas en situation d'urgence sanitaire

C'est un traitement recommandé par des confrères avec qui j'en ai discuté

C'est un traitement recommandé par des autorités médicales et/ou de la recherche

Difficulté de résister à la pression médiatique et sociale

Autre :

*Passer à la section suivante*

**Section 31**

Question complémentaire en l'absence de prescription d'HCQ

Vous est-il arrivé de ne pas prescrire de l'HCQ à l'un de vos patients alors qu'une prescription d'HCQ vous paraissait justifiée ? *

Oui *(passer à la section suivante)*

Non *(passer à la section 33)*

Je ne sais pas *(passer à la section 33)*

**Section 32**

Question complémentaire en l'absence de prescription d'HCQ

Dans ce cas, est-ce ? *

une décision prise dans un autre service que vous n'avez pas contredite

une décision collégiale de votre service même si vous étiez en faveur de la prescription

une décision prise par un collègue de votre service alors que vous étiez en faveur de la prescription

autre

*Passer à la section suivante*

**Section 33**

Information de la non prescription

Avez-vous systématiquement informé vos patients des raisons pour lesquelles vous ne leur avez pas prescrit de l'HCQ ? *

Oui, Non

*Passer à la section suivante*

__________________________________________________________________________________________

**Section 34 : Questions complémentaires pour tous les participants**

Avez-vous prescrit de l'HCQ dans le cadre d'essai(s) prospectif(s) randomisé(s) ? *

Oui, Non

*Passer à la section suivante*

**Section 35**

Avez-vous prescrit les traitements suivants au cours de l'épidémie de Covid-19 ?

Hors essai thérapeutique et Lors d'essai thérapeutique

Lopinavir/ritonavir

Remdesivir

Anti IL6 (tocilizumab, sarilumab)

Anti IL1 (anakinra)

Plasma de convalescent

Corticoïdes

Autres

Aucun de ces traitements

**Section 36**

Sources d'information

Quelles ont-été vos sources d'information sur l'intérêt potentiel de l'HCQ au cours du Covid-19 ? *

Revues médicales à comités de lecture

Revues médicales non à comité de lecture

Avis de sociétés savantes

Communication institutionnelle de votre hôpital

Sites internet médicaux

Presse "grand public" (non destinés à un public médecin)

Avis de collègues hospitaliers

Réseaux sociaux

Autre

Comment estimez-vous le risque potentiel de la prise d'HCQ au cours du Covid-19 ? *

Echelle de 0 à 10 (Aucun risque ------ Risque extrêmement important)

Comment estimez-vous le bénéfice potentiel de la prise d'HCQ au cours du Covid-19 ? *

Echelle de 0 à 10 (Aucun bénéfice ------ Bénéfice extrêmement important)

Comment estimez-vous le rapport bénéfice/risque potentiel de la prise d'HCQ au cours du Covid-19 ? *

Echelle de 0 à 10 (Rapport très défavorable ------ Rapport très favorable)

*Passer à la section suivante*

**Section 37**

Questions complémentaires pour tous les participants

Si vous étiez atteint par le SARS-Cov2 (Covid-19), pensez-vous que vous prendriez vous-même de l'HCQ ? *

Oui *(passer à la section suivante)*

Non *(passer à la section 39)*

Je ne sais pas *(passer à la section 39)*

Je ne souhaite pas répondre *(passer à la section 39)*

**Section 38**

Questions complémentaires pour tous les participants

Cela serait ? *

même en cas de forme bénigne (absence d'hospitalisation)

uniquement en cas de forme grave (hospitalisation)

*Passer à la section suivante*

**Section 39**

Questions complémentaires pour tous les participants

Si vous étiez atteint par le SARS-Cov2 (Covid-19) et qu'on vous proposait de participer à un essai thérapeutique prospectif randomisé concernant l'HCQ, que feriez-vous ?

(2 options : forme bénigne, forme grave)

Je refuserais

Je refuserais probablement

J'accepterais peut-être

J'accepterais

Je ne sais pas

Je refuserais

Je refuserais probablement

J'accepterais peut-être

J'accepterais

Je ne sais pas

*Passer à la section suivante*

**Section 40**

Questions complémentaires pour tous les participants

Si l'un de vos proches était atteint par le SARS-Cov2 (Covid-19), lui conseilleriez-vous de prendre de l'HCQ ? (hors essai prospectif randomisé) *

Oui en cas de forme bénigne (absence d'hospitalisation)

Oui en cas de forme grave (hospitalisation)

Non

Je ne sais pas

*Passer à la section suivante*

**Section 41**

Questions complémentaires pour tous les participants

La question de la prescription d'HCQ au cours du Covid-19 a-t-elle été source de difficultés avec les patients et/ou leur entourage ? *

Oui *(passer à la section suivante)*

Non *(passer à la section 43)*

Je ne sais pas *(passer à la section 43)*

**Section 42**

Questions complémentaires pour tous les participants

Etait-ce parce ce que ? *

vous n'avez pas prescrit de l'HCQ alors que le patient/son entourage le souhaitait

vous avez proposé de prescrire de l'HCQ à un patient alors qu'il étaient contre

pour une autre raison

*Passer à la section suivante*

**Section 43**

Questions complémentaires pour tous les participants

Considérez-vous que la question de la prescription d'HCQ au cours du Covid-19 a été source de débats avec des confrères de votre hôpital ? *

Oui, Non, Je ne sais pas

Comment estimez-vous la place de l'HCQ dans la prise en charge thérapeutique du Covid-19 ? *

Echelle de 0 à 10 (Aucun risque ------ Risque extrêmement important)

Avez-vous consulté le site internet de la SNFMI à la recherche d'information sur le thème de l'HCQ et du Covid-19 ? *

Oui, Non, Je ne sais pas

Estimez-vous que la pression médiatique autour de l'HCQ a influencé le fait que vous prescriviez (ou pas) de l'HCQ ? *

Non *(passer à la section 45)*

Oui *(passer à la section suivante)*

**Section 44**

Questions complémentaires pour tous les participants

Est-ce dans le sens ? *

Plus de prescription

Moins de prescription

Je ne sais pas

*Passer à la section suivante*

**Section 45**

Fin du questionnaire - Evaluation - Remerciements

Merci d'indiquer si dessous si vous avez des remarques complémentaires ou des questions à formuler.

Comment évaluez-vous la qualité de ce questionnaire ? *

Echelle de 0 à 10 (très mauvaise ------ Excellente)

Nous vous remercions très chaleureusement d'avoir compléter totalement le questionnaire.
