# Supplementary material for: Outside any therapeutic trial prescription of hydroxychloroquine for hospitalized patients with covid-19 during the first wave of the pandemic: A national inquiry of prescription patterns among French hospitalists
Source: PLoS One. 2022 Jan 21;17(1):e0261843. doi: 10.1371/journal.pone.0261843 (PMC8782345; doi:10.1371/journal.pone.0261843)
Supplement: S2 Appendix — (DOCX) [file pone.0261843.s007.docx]

**S2 Appendix. Inquiry questionnaire (English)**

**Cov-Hyd Inquiry (Hyd**roxychloroquine to treat **Cov**id-19**)**

Antoine Bosquet, PH, Isabelle Mahé, PU-PH
APHP, Service de Médecine Interne, Hôpital Louis-Mourier, Colombes, France
Université de Paris

**Questionnaire**

**Section 1**

You are a senior hospital-staff physician and managed hospitalized patients infected with SARS-CoV-2, the virus responsible for coronavirus disease-2019 (Covid-19). We are asking you to respond to this inquiry on hydroxychloroquine (HCQ) prescription to treat Covid-19 patients. Responses will be treated anonymously. Unless stated otherwise, the questions concern hospitalized patients prescribed HCQ off-label outside of any therapeutic trial.

This inquiry concerns doctors who prescribed HCQ and those who did not.

Will you accept to participate in this inquiry (estimated time ~5 minutes)

Yes

No

**Section 2: Characteristics of the participants**

We thank you in advance for your participation.

Today’s date is: ____________

You are?

A woman, a man, I prefer not to specify.

You have been practicing for?

0–4 years, 5–9 years, 10–19 years, 20–29 years, ≥30 years

Your specialty is:

Internal medicine, Cardiology, Geriatrics, Hematology–Oncology, Infectiology, Vascular medicine, Pneumology, Rheumatology, Other __________

You managed Covid-19 patients in a department in which type of facility?

A public university hospital

A public non-university hospital

A private, non-profit hospital or clinic

A private, for-profit hospital or clinic

Which region are you working in? __________

Had you prescribed HCQ before the Covid-19 pandemic for another indication?

Yes

No

I don’t know

__________________________________________________________________________________________

**Section 3**

Off-label HCQ prescription procedures in the department outside a therapeutic trial

Did your department have a common HCQ prescription procedure?

Yes (*go to the next section*)

No (*go directly to section 7)*

I don’t know (*go directly to section 7)*

**Section 4**

Procedures for HCQ prescription

In this context, the procedure is

Never prescribe HCQ in the department (*go to section 7)*

To prescribe HCQ to all hospitalized Covid-19 patients (*go to section 6)*

To prescribe HCQ to certain hospitalized Covid-19 patients meeting predefined criteria (*go to section 6)*

To prescribe HCQ only to those patients requesting it (*go to section 6)*

To leave the decision to prescribe HCQ to the discretion of each practitioner (*go to section 7)*

To collegially discuss the HCQ therapeutic option case-by-case (*go to the next section)*

I don’t know (*go to section 7)*

**Section 5**

HCQ prescription procedures

Collegial discussion was held:

During a staff meeting, during visits at the patient’s bedside, by telephone, others _______

(*go to section 7)*

**Section 6**

Application of the collegial decision to prescribe HCQ

Should a collegial decision be reached to prescribe HCQ to one of your patients, did you indeed prescribe HCQ for your patient?

Always, often, sometimes, rarely, never

*(go to the next section)*

**Section 7**

Procedure for informing the patient

Does your department have an established procedure for informing Covid-19 patients about HCQ prescription to treat their infection?

No (*go to section 9)*

Yes, targeting only patients for whom HCQ was prescribed (*go to the next section)*

Yes, targeting all patients for whom an HCQ-use indication was retained or not (*go to the next section)*

I don’t know (*go to section 9)*

**Section 8**

Procedure for informing the patient (complementary question should such a protocol exist)

In what form is the information prestented?

Written

Oral

*Go to the next section*

*__________________________________________________________________________________________*

**Section 9**

HCQ prescription for hospitalized Covid-19 patients

Have you previously prescribed HCQ to one or more Covid-19 patients that you managed (outside of a therapeutic trial)?

Yes (*go to the next section)*

*Non* (*go to section 26)*

I don’t know (*go to section 26)*

**Section 10**

HCQ prescription for hospitalized Covid-19 patients

Was HCQ started in one or several patients that you managed at that time?

Yes (*go to the next section)*

No (*go to section 17)*

**Section 11**

HCQ prescription for hospitalized Covid-19 patients

In this context, who started the discussion to potentially prescribe HCQ?

You, the patient, the patient’s entourage, a colleague, the departmental procedure in force, a collegial decision,

other _________

*go to the next section*

**Section 12**

HCQ prescription for hospitalized Covid-19 patients

Was the final decision to prescribe HCQ or not…

Collegial, individual?

*go to the next section*

**Section 13**

HCQ prescription for hospitalized Covid-19 patients

Did you prescribe HCQ (excluding contraindications)?

To all your patients (*go to section 16)*

Only certain patients among them ((*go to the next section)*

**Section 14**

HCQ prescription for hospitalized Covid-19 patients

In this context, did you prescribe HCQ based on the patient’s autonomy?

Yes, primarily for autonomous patients

Yes, primarily for non-autonomous patients

No, not according to autonomy status

I don’t know

*go to the next section*

Did you prescribe HCQ according to the patient’s comorbidities (diabetes, vascular disease, overweight/obesity, COPD …)?

Yes, primarily for patients with comorbidities

Yes, primarily for patients without comorbidities

No, independently of their comorbidities

I don’t know

Did you prescribe HCQ according to the patient’s intensive care status or not?

Yes, primarily for intensive care patients

Yes, primarily for patients not in intensive care

No, independently of their intensive care status

I don’t know

Did you prescribe HCQ according the patient’s age?

Yes, No, I don’t know

*go to the next section*

Did you prescribe HCQ according to the patient’s Covid-19 characteristics (severity, according to the time of symptom onset, progressive disease)?

Yes, (*go to the next section*)

No, (*go to section 16)*

I don’t know (*go to section 16)*

**Section 15**

HCQ prescription for hospitalized Covid-19 patients

In this case, Covid-19 was:

An early stage (for example, less than 7 days since symptom onset)

An intermediate stage (for example, between days 7 and 10 since symptom onset)

A later stage (for example, more than 10 days after symptom onset)

A benign form

An intermediate severity form

A serious form

Tending to worsen

Tending to stabilize

Tending to regress

I don’t know

*go to the next section*

**Section 16**

Criteria for prescribing HCQ to treat Covid-19

What motivated you to prescribe HCQ to your Covid-19 patients? Several responses are possible

It is an old drug with a known, favorable safety profile

Its prescription was made possible by a health ministry decree

You applied the recommendation of the collegial decision

Requested by the patient or his/her entourage

Covid-19 is a potentially very serious disease

HCQ seems to be effective against Covid-19

HCQ is an inexpensive and available drug

It was the only therapeutic option available (no alternative)

Fear of medical–legal consequences

Fear of the how I would be viewed by my departmental colleagues and my hospital

HCQ efficacy against Covid-19 was not certain but usual rules for drugs are not applicable during a public health emergency

It was treatment recommended by colleagues with whom I discussed its use

It was treatment recommended by “medical authorities”

It seemed difficult to resist media and/or societal pressure

The patient had already taken HCQ for another indication with good tolerance

Do you have another reason? If yes, please specify _______

*go to section 18*

__________________________________________________________________________________________

**Section 17**

Reasons for not using HCQ during treatment of Covid-19

For what reasons did you not start HCQ at your own initiative for one of your patients during the Covid-19 epidemic (outside a therapeutic trial)?

No indication according to currently available medical/scientific data

You didn’t even consider its prescription

No collegial or organized discussion in your department

You are opposed to off-label prescription

You deem it unethical to prescribe a non-validated drug outside therapeutic trials

Absence of official recommendations supporting HCQ prescription (Learned Societies, Academy of Medicine, physician associations…)

Fear of medical–legal consequences

Fear that HCQ could contribute to Covid-19 worsening

Fear of potential adverse events

Refusal of the patient or his/her entourage

Fear of the reactions or opinions of your colleagues

I didn’t intervene in the specific Covid-19 management of your patients

None of your patients met the criteria for HCQ prescription established in your department

Other _________

*go to the next section*

__________________________________________________________________________________________

**Section 18**

Transfer of a patient taking HCQ into your unit

If a patient taking HCQ is transferred to your unit, what do you do about HCQ prescription (in the absence of contraindications)?

Continue HCQ according to the modalities initiated by colleagues

Continue HCQ after confirming the patient’s and/or his/er entourage’s (relative/close

friend/caregiver/designated referent/legal guardian) agreement

Again, discuss the indication for HCQ

Stop HCQ after confirming the patient’s and/or his/er entourage’s (relative/close friend/caregiver/designated

referent/legal guardian) agreement

Stop HCQ because you disagree with its use to treat Covid-19

*go to the next section*

**Section 19**

Complementary questions concerning HCQ prescription to treat Covid-19

What is the HCQ prescription protocol in your department (outside therapeutic trial)?

Use of an induction dose

Prescribe as monotherapy

Prescribe in combination with azithromycin

I don’t know

None of the above

Have you observed adverse events linked to HCQ use in your patients?

Yes, (*go to the next section)*

No, (*go to section 21)*

I don’t know (*go to section 21)*

**Section 20**

HCQ adverse events

What percentage of your patients were affected? (drop-down menu)

What adverse events did you observe?

Corrected QT prolongation

Cardiac rhythm disorders (torsades de pointe, ventricular tachycardia)

Gastrointestinal disorders (abdominal pain, nausea, diarrhea, vomiting

Benign cutaneous lesions

Headaches

Severe allergic manifestation (Stevens–Johnson, Lyell ...)

Others ___________

I don’t know

Did they lead you to stop HCQ?

Yes, No, I don’t know

Did they engender any of the following consequences for the patient?

Prolongation of the hospital stay

Transfer to another department

Sequelae

Death

I don’t know

*go to the next section*

**Section 21**

HCQ prescription seems unjustified to you

Did you prescribe HCQ to a patient despite this usage appearing unjustified to you?

Yes, (*go to the next section)*

Non, (*go to section 23)*

I don’t know (*go to section 23)*

*go to the next section*

**Section 22**

HCQ prescription seems unjustified to you

In this case, was it:

A prescription started in another department that you continued

A prescription decided in your department during a collegial discussion

A prescription decided in your department by a colleague

I don’t know

*go to the next section*

**Section 23**

Complementary questions for HCQ prescribers (therapeutic trial)

How do you estimate the stress caused by prescribing HCQ (to treat Covid-19), which is a drug whose benefit/risk ratio has not been established according to standard medicine/science-based criteria?

On a Likert scale of 0–10 (no stress… maximum imaginable stress) ____

Concerning your HCQ prescriptions (therapeutic trial), have you planned to share your experience (article, congress…)?

No, (*go to section 25)*

Yes (*go to the next section)*

I don’t know (*go to section 25)*

**Section 24**

Complementary questions for HCQ prescribers outside a therapeutic trial

Did you plan à priori to do so before starting your prescriptions?

Yes

No

I don’t know

In this case, in what form?

Case report

Retrospective cohort study

An evaluation of professional practices (EPP)

If you plan a retrospective cohort study, will it include a control group?

I do not plan a cohort study

Yes

No

*go to the next section*

**Section 25**

Modification of HCQ-prescription attitudes

You prescribed HCQ to treat Covid-19. Has your approach to this prescription changed during the pandemic?

No

Yes, in that you stopped prescribing

Yes, in that prescriptions were decreased

Yes, in that prescription were increased

Yes, in that you finally started to prescribe HCQ

I don’t know

(*go to section 34)*

_________________________________________________________________________________________

**Section 26**

No HCQ prescription for Covid-19

For what reason(s) did you not prescribe HCQ during the Covid-19 pandemic (outside a therapeutic trial)?

No indication among those currently available based on medical/science data

I didn’t ask myself the whether or not to prescribe it

No collegial discussion was organized in your department

You are opposed to prescribing off-label

You consider it unethical to prescribe (outside a therapeutic trial) a drug that is not validated

Absence of official recommendations supporting HCQ prescription (Learned Societies, Academy of Medicine,

physicians’ associations)

Fear of medical–legal consequences

Refusal by the patients or his/her entourage

Fear of the reactions of opinions of my colleagues

Fear that HCQ might aggravate Covid-19

Fear of potential adverse events

You are not involved in the specific Covid-19 management of your patients

None of your patients met the criteria established in your department by you or collegially

Other ________

*go to the next section*

**Section 27**

No HSQ prescription for Covid-19

Were all of your patients included in a therapeutic trial?

Yes, (*go to the next section)*

No (*go to section 31)*

**Section 28**

Inclusion in a therapeutic trial

If all your patients had not been enrolled in clinical trials, would you have possibly prescribed HCQ?

Yes (*go to section 30)*

Non (*go to the next section)*

I don’t know (*go to section 34)*

**Section 29**

I would not have prescribed HCQ

Why?

No indication according to the available medicine/science-based data

No collegial discussion was organized in your department

You are opposed to prescribing off-label

You consider it unethical to prescribe (outside therapeutic trials) a drug that is not validated

No official recommendations supporting HCQ prescription (Learned Societies, Academy of Medicine, physicians’ associations)

Fear of medical–legal consequences

Refusal by the patients or his/her entourage

Fear of the reactions of opinions of my colleagues

Fear that HCQ might aggravate Covid-19

Fear of potential adverse events

You are not involved in the specific Covid-19 management of your patients

Other __________

(*go to section 31)*

**Section 30**

I would have prescribed HCQ.

You would have prescribed HCQ to your Covid-19 patients. For what reason(s)? Several responses are possible.

It is an old drug with a known, favorable safety profile

Its prescription was made possible by a health ministry decree

You would only have applied the recommendation of the collegial decision

If requested by the patient or his/her entourage

Covid-19 is a potentially very serious disease

HCQ seems to be effective against Covid-19

HCQ is an inexpensive and available drug

It was the only therapeutic option available (no alternative)

Fear of medical–legal consequences

Fear of the how I would be viewed by my departmental colleagues and my hospital

HCQ efficacy during Covid-19 was not certain but usual rules for medications are not applicable during a public health emergency

It is a treatment recommended by colleagues with whom I discussed its use

It is a treatment recommended by “medical authorities” and/or research findings

It seemed difficult to resist media and/or societal pressure

Do you have another reason? If yes, please specify _________________

*go to the next section*

**Section 31**

Complementary question in the absence of HCQ prescription

Did you ever not prescribe HCQ to a patient despite its prescription seeming justified?

Yes (*go to the next section)*

No (*go to section 33)*

I don’t know (*go to section 33)*

**Section 32**

Complementary question in the absence of HCQ prescription

In this case, was it:

A decision made in another department that you did not contradict

A collegial decision made in your department, even though you supported its prescription

A decision made by a colleague in your department even though you supported its prescription

Other __________

*go to the next section*

**Section 33**

Information concerning HCQ non-prescription

Did you systematically inform your patients the reasons why you did not prescribe HCQ for their Covid-19?

Yes, No

*go to the next section*

__________________________________________________________________________________________

**Section 34**

Complementary questions for all participants

Did you prescribe HCQ in the framework of a therapeutic trial)?

Yes, No

*go to the next section*

**Section 35**

Have you prescribed any of the following treatments during the Covid-19 pandemic?

Outside and within a therapeutic trial

Lopinavir/ritonavir

Remdesivir

Anti-IL6 (tocilizumab, sarilumab)

Anti-IL1 (anakinra)

Convalescent plasma

Corticosteroids

Others ___________

None of the above

**Section 36**

Sources of information

What were your sources of information concerning the potential contribution of HCQ in treating Covid-19?

Peer-reviewed medical journals

Non-peer–reviewed journals

Recommendations of Learned Societies

Institutional information sheets in your hospital

Medical websites

General public media/press (not targeting a medical public)

Advice of hospital colleagues

Social networks

Other ___________

How did you estimate the potential risk of HCQ use in a Covid-19 patient?

On a Likert scale of 0…10 (No risk … Extremely high risk) ____

How did you estimate the potential benefit of HCQ use in a Covid-19 patient?

On a Likert scale of 0…10 (No benefit … extremely high benefit) ____

How did you estimate the benefit/risk ratio of HCQ use in a Covid-19 patient?

On a Likert scale of 0…10 (Very unfavorable ratio … Highly favorable ratio) ____

**Section 37**

Complementary questions for all participants

If you were infected with SARS-CoV-2 (Covid-19), do you think you would take HCQ?

Yes (*go to the next section)*

No (*go to section 39)*

I don’t know (*go to section 39)*

I do not want to respond (*go to section 39)*

**Section 38**

Complementary questions for all participants

Would you consider it:

Even for a benign form (not requiring hospitalization)

Only for a severe form (hospitalization)

*go to the next section*

**Section 39**

Complementary questions for all participants

If you were infected with SARS-CoV-2 (Covid-19), and participated in a prospective, randomized trial on HCQ was offered, what would you do?

(2 options: benign or severe form)

I would refuse

I would probably refuse

I might accept

I would accept

I don’t know

*go to the next section*

**Section 40**

Complementary questions for all participants

If one of your relatives was infected with SARS-CoV-2 (Covid-19), would you recommend that he/she take HCQ (outside a therapeutic trial)?

Yes, for a benign form (not requiring hospitalization)

Yes, for a severe form (hospitalization)

No

I don’t know

*go to the next section*

**Section 41**

Complementary questions for all participants

Was the question of whether or not to prescribe HCQ a source of difficulties with patients and/or their entourage?

Yes (*go to the next section)*

No (*go to section 43)*

I don’t know (*go to section 43)*

**Section 42**

Complementary questions for all participants

Was it because:

You did not prescribe HCQ despite it being wanted by the patient and/or his/her entourage

You suggested HCQ and the patient was opposed

For another reason _____________

*go to the next section*

**Section 43**

Complementary questions for all participants

Do you think that the question of prescribing HCQ to treat Covid-19 was a source of debates with your hospital colleagues?

Yes, No, I don’t know

How do you estimate the place of HCQ in the therapeutic management of Covid-19?

On a Likert scale of 0…10 (No risk … Extremely important risk) _____

Did you consult the French National Société of Internal Medicine website looking for information on the subject of HCQ and Covid-19?

Yes, No, I don’t know

Do you think that the media pressure surrounding HCQ influenced your prescribing HCQ (or not)?

No, (*go to section 45)*

Yes (*go to the next section)*

**Section 44**

Complementary questions for all participants

Was it in the sense that you:

Prescribed it more

Prescribed it less

I don’t know

*go to the next section*

**Section 45**

End of the questionnaire – Evaluation – Acknowledgments

Please indicate below if you have any additional remarks or questions.

How do you evaluate the quality of this questionnaire?

On a Likert scale of 0…10 (very bad … Excellent) ____

We want to sincerely thank you for having completed the entire questionnaire. If you want to contact me, you can do so via email: [antoine.bosquet@aphp.fr](mailto:antoine.bosquet@aphp.fr).
