# Supplementary material for: Outside any therapeutic trial prescription of hydroxychloroquine for hospitalized patients with covid-19 during the first wave of the pandemic: A national inquiry of prescription patterns among French hospitalists
Source: PLoS One. 2022 Jan 21;17(1):e0261843. doi: 10.1371/journal.pone.0261843 (PMC8782345; doi:10.1371/journal.pone.0261843)
Supplement: S3 Appendix — (DOCX) [file pone.0261843.s008.docx]

**S3 Appendix. Checklist for Reporting Results of Internet E-Surveys (CHERRIES)***

| **Item category** | **Checklist item** | **Page no.** | **Description** |
| --- | --- | --- | --- |
| Design | Survey design | 7 | A national French inquiry of internal medicine or infectious disease hospitalists managing covid-19 patients in France |
| IRB (Institutional Review Board) approval and informed consent process | Ethics approval | 8 | This study was declared to the French Computer Watchdog Commission (French acronym CNIL). The agreement of conformity of the study was obtained from the CNIL on April 20, 2020 (no. 2217633 v 0). |
|  | Informed consent | 7 | All participating physicians provided informed written consent prior to gaining access to the questionnaire. |
|  | Date protection | 7-9 | Data from all individual questionnaires were extracted from the Google Form file, transferred to Microsoft Excel, and then converted into a file to create the database for analysis using SAS v9.4 (SAS Institute Inc, Cary, NC) statistical software package. |
| Development and pre-testing |  | 7 | The first versions of the questionnaire were tested on departmental hospitalists (IM, LP, LA, JC, SD) to evaluate comprehension of the items and the time needed to complete the inquiry. |
| Recruitment process and description  of the sample having access  to the questionnaire | Open survey versus closed survey | 7 | Closed inquiry: it was administered electronically to 1879 French hospitalists. |
|  | Contact mode | 7 | Participating physicians received e-mail invitations to participate that included a link to the inquiry’s online portal (an electronic questionnaire via Google Form©). |
|  | Advertising the survey | 7 | No ads were placed to seek participants. |
| Survey administration | Web/E-mail | 7 | Participating physicians received e-mail invitations to participate that included a link to the inquiry’s online portal (an electronic questionnaire via Google Form©). |
|  | Context | 7 | The inquiry was administered to and completed electronically by 1879 hospitalists managing covid-19 patients in a French internal medicine or infectious disease department and entered in the directories of French hospitals or members of the French Infectious Diseases Society. |
|  | Mandatory/voluntary | 7 | Voluntary inquiry |
|  | Incentives | N/A | No incentives |
|  | Time/Date | 7 | A link to the electronic questionnaire was sent by e-mail starting May 1, 2020, with reminders sent at 1-week intervals, and closure May 25, 2020. |
|  | Randomization of items or questionnaires | 7 | Items were not randomized. |
|  | Adaptive questioning | 7 | The inquiry used response-adapted questioning (certain items, or only conditionally displayed based on responses to other items) to limit the number and complexity of the questions. |
|  | Number of Items | 7 | The questionnaire consists of 68 items: 25 systematically submitted to all participants and 43 according to the previous answers. Hence, the number of items varies from 41 to 62 for HCQ prescribers, from 37 to 53 for non-prescribers. |
|  | Number of screens (pages) | 7 | 69 screens |
|  | Completeness check | 7 | We did not perform consistency or completeness checks before the questionnaire was submitted. All but four of the items required responses. Most items (39/68) provided a non-response option, such as “I don’t know” (n=30) or an option “other response” (n=9). |
|  | Review step | N/A | Respondents were not able to review and change their preceding responses (eg, through a return button). |
| Response rates | Unique site visitor | N/A | We did not use a technique based on IP addresses or cookies to ensure the anonymity of the inquiry. |
|  | View rate | N/A |  |
|  | Participation rate | 7, 9 | To calculate the participation rate (ie, 400/1879 physicians), we chose the number of responses (n=400) as the numerator and the number of physicians offered participation in the inquiry (n=1879) as the denominator, not taking into consideration duplicates (n=80) and the doctors who did not receive the e-mails because of erroneous addresses (n=127) (ie, 1879 – 80 – 127=1752). |
|  | Completion rate | 7, 9 | This rate was calculated as above for the participation rate. |
| Preventing multiple entries from the same individual | Cookies used | N/A | No cookies used. |
|  | IP check | N/A | No IP checking. |
|  | Log file analysis | N/A | No log-file analysis. |
|  | Registration | N/A | No registration. |
| Analysis | Handling of incomplete questionnaires | N/A | All questionnaires submitted were fully completed and analyzed. |
|  | Questionnaires submitted with  an atypical timestamp | N/A |  |
|  | Statistical correction | N/A |  |

*Eysenbach G. Improving the quality of Web surveys: the Checklist for Reporting Results of Internet E-Surveys (CHERRIES). J Med Internet Res. 2004;6(3).

N/A: not applicable.
